# Supplementary material for: Iterative improvement in the automatic modular design of robot swarms
Source: PeerJ Comput Sci. 2020 Dec 7;6:e322. doi: 10.7717/peerj-cs.322 (PMC7924708; doi:10.7717/peerj-cs.322)
Supplement: Supplemental Information 3 [file peerj-cs-06-322-s003.zip › argos3/doc/api/standalone/a00495_source.html]

ARGoS: plugins/robots/generic/control\_interface/ci\_differential\_steering\_sensor.h Source File


- Main Page
- Related Pages
- Namespaces
- Classes
- Files

- File List
- File Members

# plugins/robots/generic/control\_interface/ci\_differential\_steering\_sensor.h

Go to the documentation of this file.

```
00001 
00007 #ifndef CCI_DIFFERENTIAL_STEERING_SENSOR_H
00008 #define CCI_DIFFERENTIAL_STEERING_SENSOR_H
00009 
00010 namespace argos {
00011    class CCI_DifferentialSteeringSensor;
00012 }
00013 
00014 #include <argos3/core/control_interface/ci_sensor.h>
00015 
00016 namespace argos {
00017 
00018    class CCI_DifferentialSteeringSensor : public CCI_Sensor {
00019 
00020    public:
00021 
00022       struct SReading {
00023          Real VelocityLeftWheel;
00024          Real VelocityRightWheel;
00025          Real CoveredDistanceLeftWheel;
00026          Real CoveredDistanceRightWheel;
00027          Real WheelAxisLength;
00028          
00029          SReading() :
00030             VelocityLeftWheel(0.0f),
00031             VelocityRightWheel(0.0f),
00032             CoveredDistanceLeftWheel(0.0f),
00033             CoveredDistanceRightWheel(0.0f),
00034             WheelAxisLength(0.0f) {}
00035          
00036          SReading(Real f_velocity_left_wheel,
00037                   Real f_velocity_right_wheel,
00038                   Real f_covered_distance_left_wheel,
00039                   Real f_covered_distance_right_wheel,
00040                   Real f_wheel_axis_length) :
00041             VelocityLeftWheel(f_velocity_left_wheel),
00042             VelocityRightWheel(f_velocity_right_wheel),
00043             CoveredDistanceLeftWheel(f_covered_distance_left_wheel),
00044             CoveredDistanceRightWheel(f_covered_distance_right_wheel),
00045             WheelAxisLength(f_wheel_axis_length) {
00046          }
00047 
00048       };
00049 
00053       CCI_DifferentialSteeringSensor() {}
00054 
00058       virtual ~CCI_DifferentialSteeringSensor() {}
00059 
00064       const SReading& GetReading() const;
00065 
00066 #ifdef ARGOS_WITH_LUA
00067       virtual void CreateLuaState(lua_State* pt_lua_state);
00068 
00069       virtual void ReadingsToLuaState(lua_State* pt_lua_state);
00070 #endif
00071 
00072    protected:
00073 
00074       SReading m_sReading;
00075    };
00076 
00077 }
00078 
00079 #endif
```

---

Generated on 10 Jul 2018 for ARGoS by 
 1.6.1
